# 3-Methyl-5-phenyl-1-(3-phenyl­isoquinolin-1-yl)-1*H*-pyrazole

**DOI:** 10.1107/S160053680905572X

**Published:** 2010-01-16

**Authors:** F. Nawaz Khan, P. Manivel, Sriramakrishnaswamy Kone, Venkatesha R. Hathwar, Seik Weng Ng

**Affiliations:** a Chemistry Division, School of Advanced Sciences, VIT University, Vellore 632014, Tamil Nadu, India; b Solid State and Structural Chemistry Unit, Indian Institute of Science, Bangalore 560012, Karnataka, India; c Department of Chemistry, University of Malaya, 50603 Kuala Lumpur, Malaysia

## Abstract

The title compound, C_25_H_19_N_3_, is composed of an aryl-substituted pyrazole ring connected to an aryl-substituted isoquinoline ring system with a dihedral angle of 52.7 (1)° between the pyrazole ring and the isoquinoline ring system. The dihedral angle between the pyrazole ring and the phenyl ring attached to it is 27.4 (1)° and the dihedral angle between the isoquinoline ring system and the phenyl ring attached to it is 19.6 (1)°.

## Related literature

For medicinal applications of hydrazine derivatives, see: Broadhurst *et al.* (2001 ▶).
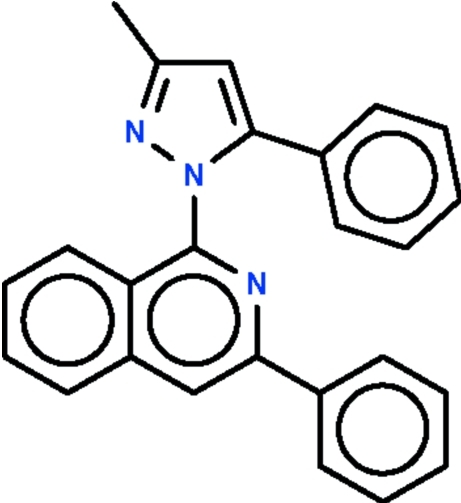


## Experimental

### 

#### Crystal data

C_25_H_19_N_3_
*M* *_r_* = 361.43
Orthorhombic, 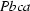

*a* = 10.9610 (9) Å
*b* = 16.8078 (13) Å
*c* = 21.3118 (17) Å
*V* = 3926.3 (5) Å^3^
*Z* = 8
Mo *K*α radiation
μ = 0.07 mm^−1^
*T* = 290 K
0.42 × 0.23 × 0.20 mm

#### Data collection

Bruker SMART area-detector diffractometer
26302 measured reflections
3452 independent reflections
2031 reflections with *I* > 2σ(*I*)
*R*_int_ = 0.091

#### Refinement

*R*[*F* ^2^ > 2σ(*F* ^2^)] = 0.059
*wR*(*F* ^2^) = 0.172
*S* = 1.04
3452 reflections
254 parameters
H-atom parameters constrained
Δρ_max_ = 0.16 e Å^−3^
Δρ_min_ = −0.16 e Å^−3^

### 

Data collection: *SMART* (Bruker, 2004 ▶); cell refinement: *SAINT* (Bruker, 2004 ▶); data reduction: *SAINT*; program(s) used to solve structure: *SHELXS97* (Sheldrick, 2008 ▶); program(s) used to refine structure: *SHELXL97* (Sheldrick, 2008 ▶); molecular graphics: *X-SEED* (Barbour, 2001 ▶); software used to prepare material for publication: *publCIF* (Westrip, 2010 ▶).

## Supplementary Material

Crystal structure: contains datablocks global, I. DOI: 10.1107/S160053680905572X/bt5159sup1.cif

Structure factors: contains datablocks I. DOI: 10.1107/S160053680905572X/bt5159Isup2.hkl

Additional supplementary materials:  crystallographic information; 3D view; checkCIF report

## Figures and Tables

**Fig. 1.**
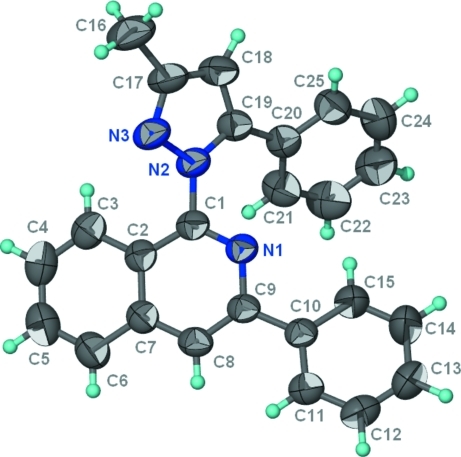
Anisotropic displacement ellipsoid plot (Barbour, 2001) of C25H19N3 at the 50% probability level; hydrogen atoms are drawn as spheres of arbitrary radius.

## supplementary crystallographic information

### Experimental

3-Phenylisoquinolin-1-ylhydrazine (2.35 g, 10 mmol) and 1-phenylbutane-1,3-dione (1.62 g, 10 mmol) were dissolved in ethanol (30 ml). The solution was heated for 12 h under a nitrogen atmosphere. The reaction was quenched with water; the compound was extracted with ethyl acetate. The ethyl acetate phase was washed with water, dried, concentrated and purified by column chromatography to yield a white powder. Crystals were   obtained upon recrystallization from dichloromethane.

### Refinement

Hydrogen atoms were placed in calculated positions (C–H 0.93–0.96 Å) and were included in the refinement in the riding model approximation, with *U*(H) set to 1.2–1.5*U*_eq_(C).

### Crystal data

**Table:**

C_25_H_19_N_3_	*F*(000) = 1520
*M**_r_* = 361.43	*D*_x_ = 1.223 Mg m^−^^3^
Orthorhombic, *P**b**c**a*	Mo *K*α radiation, λ = 0.71073 Å
Hall symbol: -P 2ac 2ab	Cell parameters from 1667 reflections
*a* = 10.9610 (9) Å	θ = 2.4–19.5°
*b* = 16.8078 (13) Å	µ = 0.07 mm^−^^1^
*c* = 21.3118 (17) Å	*T* = 290 K
*V* = 3926.3 (5) Å^3^	Block, colorless
*Z* = 8	0.42 × 0.23 × 0.20 mm

### Data collection

**Table:**

Bruker SMART area-detector diffractometer	2031 reflections with *I* > 2σ(*I*)
Radiation source: fine-focus sealed tube	*R*_int_ = 0.091
graphite	θ_max_ = 25.0°, θ_min_ = 1.9°
φ and ω scans	*h* = −13→13
26302 measured reflections	*k* = −19→19
3452 independent reflections	*l* = −25→25

### Refinement

**Table:**

Refinement on *F*^2^	Primary atom site location: structure-invariant direct methods
Least-squares matrix: full	Secondary atom site location: difference Fourier map
*R*[*F*^2^ > 2σ(*F*^2^)] = 0.059	Hydrogen site location: inferred from neighbouring sites
*wR*(*F*^2^) = 0.172	H-atom parameters constrained
*S* = 1.04	*w* = 1/[σ^2^(*F*_o_^2^) + (0.0822*P*)^2^ + 0.3609*P*] where *P* = (*F*_o_^2^ + 2*F*_c_^2^)/3
3452 reflections	(Δ/σ)_max_ = 0.001
254 parameters	Δρ_max_ = 0.16 e Å^−^^3^
0 restraints	Δρ_min_ = −0.16 e Å^−^^3^

### Fractional atomic coordinates and isotropic or equivalent isotropic displacement parameters (Å^2^)

**Table:**

	*x*	*y*	*z*	*U*_iso_*/*U*_eq_	
N1	0.42399 (17)	0.56804 (11)	0.65341 (8)	0.0632 (5)	
N2	0.58627 (17)	0.61748 (12)	0.59624 (9)	0.0707 (6)	
N3	0.70861 (18)	0.60123 (14)	0.60212 (10)	0.0846 (7)	
C1	0.5164 (2)	0.61722 (15)	0.65309 (11)	0.0634 (6)	
C2	0.5528 (2)	0.66672 (14)	0.70362 (11)	0.0633 (6)	
C3	0.6493 (2)	0.72259 (17)	0.70207 (14)	0.0799 (8)	
H3	0.6940	0.7293	0.6654	0.096*	
C4	0.6771 (3)	0.76627 (17)	0.75330 (18)	0.0925 (9)	
H4	0.7414	0.8023	0.7517	0.111*	
C5	0.6101 (3)	0.75780 (18)	0.80879 (16)	0.0963 (9)	
H5	0.6304	0.7881	0.8438	0.116*	
C6	0.5156 (3)	0.70560 (18)	0.81196 (14)	0.0876 (8)	
H6	0.4713	0.7006	0.8490	0.105*	
C7	0.4844 (2)	0.65902 (14)	0.75938 (11)	0.0681 (6)	
C8	0.3851 (2)	0.60570 (14)	0.75944 (11)	0.0691 (7)	
H8	0.3392	0.5993	0.7958	0.083*	
C9	0.3554 (2)	0.56334 (14)	0.70713 (11)	0.0617 (6)	
C10	0.2482 (2)	0.50980 (14)	0.70392 (11)	0.0635 (6)	
C11	0.1918 (2)	0.48178 (15)	0.75749 (12)	0.0745 (7)	
H11	0.2226	0.4958	0.7966	0.089*	
C12	0.0904 (3)	0.43317 (16)	0.75384 (15)	0.0858 (8)	
H12	0.0538	0.4149	0.7905	0.103*	
C13	0.0441 (3)	0.4121 (2)	0.69771 (17)	0.0999 (10)	
H13	−0.0241	0.3793	0.6955	0.120*	
C14	0.0978 (3)	0.4392 (2)	0.64406 (16)	0.1194 (12)	
H14	0.0660	0.4248	0.6052	0.143*	
C15	0.1993 (3)	0.4880 (2)	0.64698 (13)	0.0944 (9)	
H15	0.2350	0.5062	0.6101	0.113*	
C16	0.8797 (3)	0.5726 (3)	0.53211 (16)	0.1305 (13)	
H16A	0.9202	0.5639	0.5715	0.196*	
H16B	0.9171	0.6166	0.5108	0.196*	
H16C	0.8864	0.5257	0.5067	0.196*	
C17	0.7463 (2)	0.59087 (19)	0.54383 (14)	0.0888 (8)	
C18	0.6524 (3)	0.59926 (17)	0.50078 (13)	0.0849 (8)	
H18	0.6579	0.5946	0.4574	0.102*	
C19	0.5500 (2)	0.61580 (14)	0.53516 (12)	0.0688 (7)	
C20	0.4262 (2)	0.63444 (15)	0.51195 (12)	0.0700 (7)	
C21	0.3472 (3)	0.68436 (17)	0.54353 (13)	0.0869 (8)	
H21	0.3704	0.7068	0.5816	0.104*	
C22	0.2326 (3)	0.7011 (2)	0.51841 (16)	0.1031 (10)	
H22	0.1792	0.7335	0.5407	0.124*	
C23	0.1976 (3)	0.6712 (2)	0.46237 (18)	0.1081 (10)	
H23	0.1214	0.6835	0.4458	0.130*	
C24	0.2752 (3)	0.6231 (2)	0.43068 (16)	0.1117 (11)	
H24	0.2522	0.6027	0.3919	0.134*	
C25	0.3885 (3)	0.60395 (18)	0.45521 (13)	0.0887 (8)	
H25	0.4398	0.5700	0.4330	0.106*	

### Atomic displacement parameters (Å^2^)

**Table:**

	*U*^11^	*U*^22^	*U*^33^	*U*^12^	*U*^13^	*U*^23^
N1	0.0544 (11)	0.0749 (13)	0.0603 (12)	0.0075 (10)	0.0015 (10)	0.0039 (9)
N2	0.0542 (12)	0.0933 (15)	0.0646 (13)	0.0066 (11)	0.0042 (10)	0.0105 (10)
N3	0.0552 (13)	0.1212 (19)	0.0773 (15)	0.0096 (12)	0.0066 (11)	0.0139 (13)
C1	0.0534 (13)	0.0770 (16)	0.0598 (15)	0.0118 (13)	0.0008 (11)	0.0094 (12)
C2	0.0553 (14)	0.0668 (15)	0.0679 (16)	0.0089 (12)	−0.0046 (12)	0.0053 (12)
C3	0.0663 (16)	0.0801 (18)	0.093 (2)	0.0056 (15)	−0.0055 (14)	0.0093 (15)
C4	0.0795 (19)	0.0796 (19)	0.118 (3)	−0.0061 (15)	−0.0244 (19)	0.0043 (19)
C5	0.100 (2)	0.090 (2)	0.099 (3)	0.0029 (19)	−0.0241 (19)	−0.0117 (17)
C6	0.091 (2)	0.091 (2)	0.081 (2)	0.0016 (18)	−0.0062 (15)	−0.0128 (15)
C7	0.0642 (14)	0.0718 (15)	0.0682 (17)	0.0086 (13)	−0.0041 (13)	−0.0033 (13)
C8	0.0652 (15)	0.0802 (17)	0.0619 (16)	0.0128 (13)	0.0063 (12)	−0.0027 (13)
C9	0.0550 (13)	0.0701 (15)	0.0600 (15)	0.0128 (12)	0.0043 (11)	0.0010 (12)
C10	0.0557 (13)	0.0697 (15)	0.0652 (16)	0.0103 (12)	0.0054 (12)	−0.0026 (12)
C11	0.0721 (16)	0.0797 (17)	0.0717 (17)	0.0030 (14)	0.0063 (14)	0.0030 (13)
C12	0.0782 (18)	0.0835 (18)	0.096 (2)	0.0000 (15)	0.0207 (17)	0.0067 (16)
C13	0.076 (2)	0.113 (2)	0.110 (3)	−0.0224 (17)	0.0062 (18)	−0.002 (2)
C14	0.102 (2)	0.164 (3)	0.092 (2)	−0.051 (2)	−0.0043 (19)	−0.012 (2)
C15	0.0830 (18)	0.129 (3)	0.0713 (18)	−0.0221 (18)	0.0066 (15)	−0.0034 (17)
C16	0.0710 (19)	0.202 (4)	0.119 (3)	0.025 (2)	0.0287 (18)	0.014 (3)
C17	0.0685 (17)	0.121 (2)	0.0765 (19)	0.0118 (16)	0.0171 (15)	0.0141 (17)
C18	0.0853 (19)	0.106 (2)	0.0633 (16)	0.0106 (16)	0.0141 (15)	0.0110 (14)
C19	0.0733 (16)	0.0701 (16)	0.0630 (16)	0.0038 (13)	0.0045 (13)	0.0093 (12)
C20	0.0748 (16)	0.0685 (15)	0.0667 (17)	0.0027 (13)	0.0002 (14)	0.0110 (12)
C21	0.091 (2)	0.093 (2)	0.0768 (18)	0.0228 (17)	−0.0073 (15)	0.0028 (14)
C22	0.097 (2)	0.121 (3)	0.091 (2)	0.0377 (19)	−0.0039 (19)	0.0170 (18)
C23	0.084 (2)	0.144 (3)	0.097 (3)	0.017 (2)	−0.0086 (19)	0.022 (2)
C24	0.101 (2)	0.145 (3)	0.089 (2)	−0.005 (2)	−0.024 (2)	−0.004 (2)
C25	0.089 (2)	0.102 (2)	0.0748 (19)	0.0065 (17)	−0.0030 (16)	−0.0044 (15)

### Geometric parameters (Å, °)

**Table:**

N1—C1	1.308 (3)	C12—H12	0.9300
N1—C9	1.372 (3)	C13—C14	1.365 (4)
N2—C19	1.362 (3)	C13—H13	0.9300
N2—N3	1.374 (3)	C14—C15	1.383 (4)
N2—C1	1.433 (3)	C14—H14	0.9300
N3—C17	1.321 (3)	C15—H15	0.9300
C1—C2	1.418 (3)	C16—C17	1.515 (4)
C2—C7	1.411 (3)	C16—H16A	0.9600
C2—C3	1.414 (3)	C16—H16B	0.9600
C3—C4	1.351 (4)	C16—H16C	0.9600
C3—H3	0.9300	C17—C18	1.385 (4)
C4—C5	1.399 (4)	C18—C19	1.370 (3)
C4—H4	0.9300	C18—H18	0.9300
C5—C6	1.359 (4)	C19—C20	1.478 (3)
C5—H5	0.9300	C20—C25	1.377 (3)
C6—C7	1.409 (3)	C20—C21	1.381 (4)
C6—H6	0.9300	C21—C22	1.394 (4)
C7—C8	1.410 (3)	C21—H21	0.9300
C8—C9	1.362 (3)	C22—C23	1.351 (4)
C8—H8	0.9300	C22—H22	0.9300
C9—C10	1.481 (3)	C23—C24	1.355 (5)
C10—C15	1.376 (3)	C23—H23	0.9300
C10—C11	1.381 (3)	C24—C25	1.385 (4)
C11—C12	1.382 (4)	C24—H24	0.9300
C11—H11	0.9300	C25—H25	0.9300
C12—C13	1.347 (4)		
			
C1—N1—C9	117.7 (2)	C12—C13—H13	120.2
C19—N2—N3	111.61 (19)	C14—C13—H13	120.2
C19—N2—C1	130.7 (2)	C13—C14—C15	120.5 (3)
N3—N2—C1	116.34 (19)	C13—C14—H14	119.7
C17—N3—N2	104.2 (2)	C15—C14—H14	119.8
N1—C1—C2	125.8 (2)	C10—C15—C14	120.7 (3)
N1—C1—N2	114.9 (2)	C10—C15—H15	119.6
C2—C1—N2	119.3 (2)	C14—C15—H15	119.6
C7—C2—C3	118.5 (2)	C17—C16—H16A	109.5
C7—C2—C1	115.9 (2)	C17—C16—H16B	109.5
C3—C2—C1	125.6 (2)	H16A—C16—H16B	109.5
C4—C3—C2	120.7 (3)	C17—C16—H16C	109.5
C4—C3—H3	119.6	H16A—C16—H16C	109.5
C2—C3—H3	119.6	H16B—C16—H16C	109.5
C3—C4—C5	120.6 (3)	N3—C17—C18	112.2 (2)
C3—C4—H4	119.7	N3—C17—C16	118.9 (3)
C5—C4—H4	119.7	C18—C17—C16	128.9 (3)
C6—C5—C4	120.5 (3)	C19—C18—C17	106.0 (2)
C6—C5—H5	119.7	C19—C18—H18	127.0
C4—C5—H5	119.7	C17—C18—H18	127.0
C5—C6—C7	120.3 (3)	N2—C19—C18	106.0 (2)
C5—C6—H6	119.9	N2—C19—C20	125.7 (2)
C7—C6—H6	119.9	C18—C19—C20	128.1 (2)
C8—C7—C2	118.0 (2)	C25—C20—C21	117.8 (3)
C8—C7—C6	122.7 (2)	C25—C20—C19	119.4 (2)
C2—C7—C6	119.3 (2)	C21—C20—C19	122.8 (2)
C9—C8—C7	121.1 (2)	C20—C21—C22	120.0 (3)
C9—C8—H8	119.5	C20—C21—H21	120.0
C7—C8—H8	119.5	C22—C21—H21	120.0
C8—C9—N1	121.5 (2)	C23—C22—C21	121.4 (3)
C8—C9—C10	123.0 (2)	C23—C22—H22	119.3
N1—C9—C10	115.5 (2)	C21—C22—H22	119.3
C15—C10—C11	117.6 (2)	C22—C23—C24	118.9 (3)
C15—C10—C9	120.8 (2)	C22—C23—H23	120.5
C11—C10—C9	121.6 (2)	C24—C23—H23	120.5
C12—C11—C10	121.0 (2)	C23—C24—C25	120.9 (3)
C12—C11—H11	119.5	C23—C24—H24	119.5
C10—C11—H11	119.5	C25—C24—H24	119.5
C13—C12—C11	120.6 (3)	C20—C25—C24	120.9 (3)
C13—C12—H12	119.7	C20—C25—H25	119.5
C11—C12—H12	119.7	C24—C25—H25	119.5
C12—C13—C14	119.6 (3)		
			
C19—N2—N3—C17	0.9 (3)	N1—C9—C10—C11	161.5 (2)
C1—N2—N3—C17	169.0 (2)	C15—C10—C11—C12	0.4 (4)
C9—N1—C1—C2	0.0 (3)	C9—C10—C11—C12	178.7 (2)
C9—N1—C1—N2	178.25 (18)	C10—C11—C12—C13	−0.1 (4)
C19—N2—C1—N1	42.3 (3)	C11—C12—C13—C14	−0.1 (5)
N3—N2—C1—N1	−123.0 (2)	C12—C13—C14—C15	0.1 (5)
C19—N2—C1—C2	−139.3 (2)	C11—C10—C15—C14	−0.4 (4)
N3—N2—C1—C2	55.3 (3)	C9—C10—C15—C14	−178.7 (3)
N1—C1—C2—C7	2.4 (3)	C13—C14—C15—C10	0.2 (5)
N2—C1—C2—C7	−175.80 (18)	N2—N3—C17—C18	−0.5 (3)
N1—C1—C2—C3	−176.8 (2)	N2—N3—C17—C16	179.2 (3)
N2—C1—C2—C3	5.1 (3)	N3—C17—C18—C19	−0.1 (3)
C7—C2—C3—C4	1.6 (3)	C16—C17—C18—C19	−179.8 (3)
C1—C2—C3—C4	−179.3 (2)	N3—N2—C19—C18	−1.0 (3)
C2—C3—C4—C5	−0.8 (4)	C1—N2—C19—C18	−166.9 (2)
C3—C4—C5—C6	−0.2 (4)	N3—N2—C19—C20	−176.8 (2)
C4—C5—C6—C7	0.4 (4)	C1—N2—C19—C20	17.3 (4)
C3—C2—C7—C8	177.2 (2)	C17—C18—C19—N2	0.6 (3)
C1—C2—C7—C8	−2.0 (3)	C17—C18—C19—C20	176.3 (3)
C3—C2—C7—C6	−1.4 (3)	N2—C19—C20—C25	−156.5 (2)
C1—C2—C7—C6	179.4 (2)	C18—C19—C20—C25	28.6 (4)
C5—C6—C7—C8	−178.1 (2)	N2—C19—C20—C21	26.4 (4)
C5—C6—C7—C2	0.4 (4)	C18—C19—C20—C21	−148.5 (3)
C2—C7—C8—C9	−0.5 (3)	C25—C20—C21—C22	1.3 (4)
C6—C7—C8—C9	178.0 (2)	C19—C20—C21—C22	178.5 (2)
C7—C8—C9—N1	3.1 (3)	C20—C21—C22—C23	−2.0 (5)
C7—C8—C9—C10	−176.8 (2)	C21—C22—C23—C24	1.0 (5)
C1—N1—C9—C8	−2.8 (3)	C22—C23—C24—C25	0.6 (6)
C1—N1—C9—C10	177.05 (19)	C21—C20—C25—C24	0.3 (4)
C8—C9—C10—C15	159.6 (2)	C19—C20—C25—C24	−177.0 (3)
N1—C9—C10—C15	−20.2 (3)	C23—C24—C25—C20	−1.2 (5)
C8—C9—C10—C11	−18.6 (3)		
